# Publisher Correction: Evidence of radial Weibel instability in relativistic intensity laser-plasma interactions inside a sub-micron thick liquid target

**DOI:** 10.1038/s41598-020-70074-2

**Published:** 2020-09-30

**Authors:** Gregory K. Ngirmang, John T. Morrison, Kevin M. George, Joseph R. Smith, Kyle D. Frische, Chris Orban, Enam A. Chowdhury, W. Mel Roquemore

**Affiliations:** 1 National Academies of Sciences, Engineering, and Medicine, Washington, DC USA; 2 Innovative Scientific Solutions, Inc., Dayton, OH 45459 USA; 3 Department of Physics, Ohio State University, Columbus, OH 43210 USA; 4 Intense Energy Solutions, LLC., Plain City, OH 43064 USA; 5 Department of Material Science and Engineering, Ohio State University, Columbus, OH 43210 USA; 6 Air Force Research Laboratory, Dayton, OH 45433 USA

Correction to: *Scientific Reports* https://doi.org/10.1038/s41598-020-66615-4, published online 18 June 2020

The following supplementary files were omitted from the original version of this Article:

- ‘Supplementary Information Video 1b’
- ‘Supplementary Information Video 1c’
- ‘Supplementary Information Video 1d’
- ‘Supplementary Information Video 3’
- ‘Supplementary Information Video 4’

This has been corrected in the HTML version the article. The PDF was correct at the time of publishing.

