# Supplementary material for: Evidence of radial Weibel instability in relativistic intensity laser-plasma interactions inside a sub-micron thick liquid target
Source: Sci Rep. 2020 Jun 18;10:9872. doi: 10.1038/s41598-020-66615-4 (PMC7303142; doi:10.1038/s41598-020-66615-4)
Supplement: Supplementary file 1 — Supplementary Information. [file 41598_2020_66615_MOESM1_ESM.pdf]

Evidence of radial Weibel instability in relativistic intensity laser-plasma interactions inside a sub-micron thick liquid target, Supplementary Figures

Gregory K. Ngirmang[1,6,*], John T. Morrison[2], Kevin M. George[2], Joseph R. Smith[3], Kyle D. Frische[2], Chris Orban[3], Enam A. Chowdhury[4,5], and W.Mel Roquemore[6]

1) National Academies of Sciences, Engineering, and Medicine, Washington DC, USA
2) Innovative Scientific Solutions, Inc., Dayton, Ohio 45459, USA
3) Ohio State University, Department of Physics Columbus, Ohio 43210, USA
4) Intense Energy Solutions, LLC., Plain City, Ohio 43064, USA
5) Ohio State University, Department of Material Science and Engineering, Ohio 43210, USA
6) Air Force Research Laboratory, Dayton, Ohio 45433, USA
*ngirmang@protonmail.com

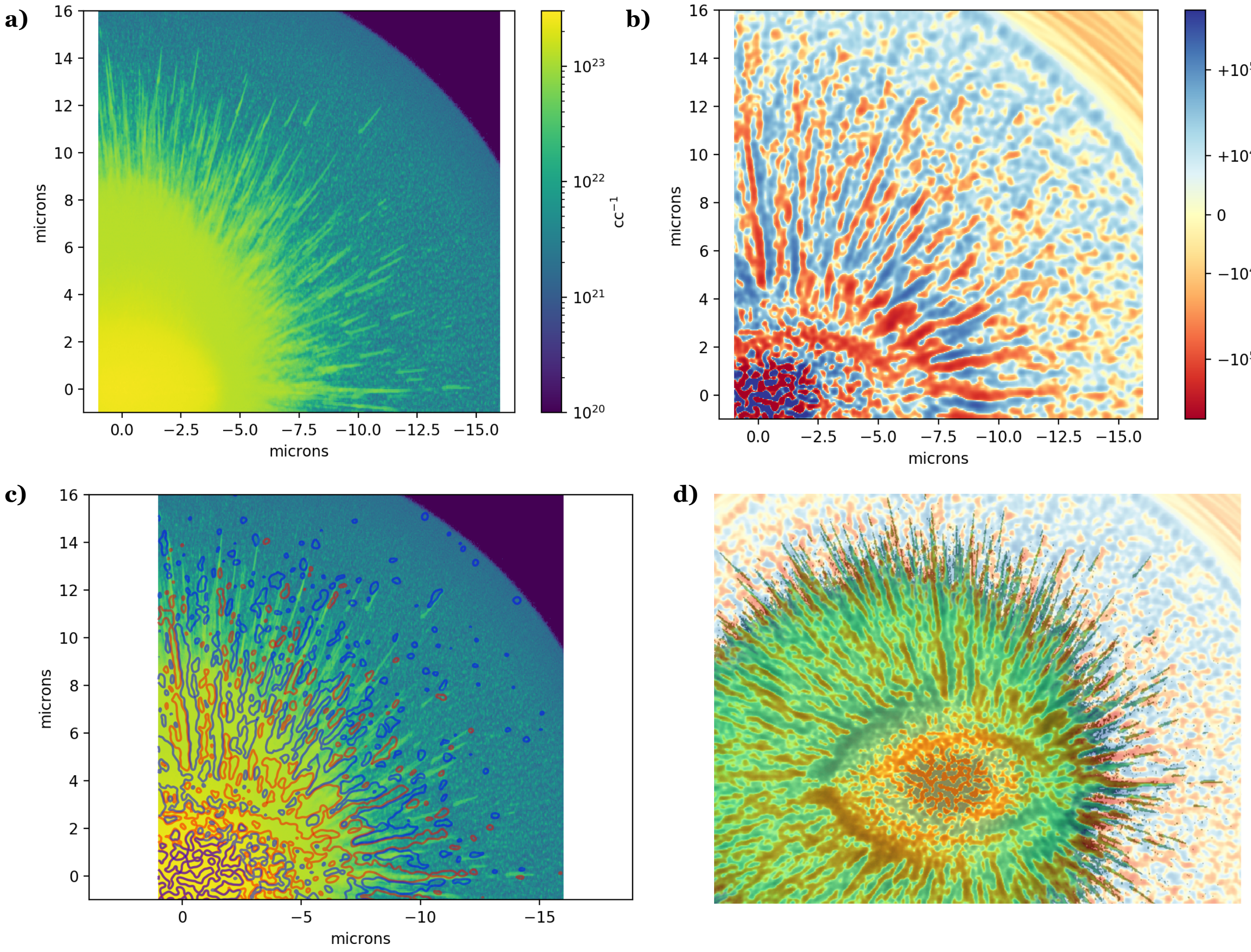

Supplementary Figure S1. Simulation data zoomed in to show detail. **a)** Free Electron density in the plane, **b)** Magnetic field component normal to the target plane. This data is from the same time step as Fig.1c) and Fig.1d) in the main article text. **c)** Demonstrates overlap between the filaments and fields by contours of the magnetic field overlaid on the electron density, with blue and red contours corresponding to $+4\times10^4$ gauss and -4×10^4 gauss respectively. **d)** Shows data from the corresponding figures in the text overlaid in transparency to again highlight correspondence between the fields and the filaments. These figures were produced using Matplotlib[1].

References

1. Hunter, J. D. Matplotlib: A 2d graphics environment. *Comput. Sci. & Eng.* **9**, 90–95, DOI: 10.1109/MCSE.2007.55 (2007).
